# Low-dose anti-VEGFR2 therapy promotes anti-tumor immunity in lung adenocarcinoma by down-regulating the expression of layilin on tumor-infiltrating CD8^+^T cells

**DOI:** 10.1007/s13402-022-00718-0

**Published:** 2022-10-19

**Authors:** Biaolong Yang, Biaolong Deng, Xiao-Dong Jiao, Bao-Dong Qin, Yi Lu, Weiqi Zhang, Yixian Guo, Shiqi Chen, Dan Li, Bin Li, Yuan-Sheng Zang

**Affiliations:** 1 Department of Oncology, Changzheng Hospital, Naval Medical University, 200003 Shanghai, China; 2 Department of Immunology and Microbiology, Shanghai Institute of Immunology, Shanghai Jiao Tong University School of Medicine, 200025 Shanghai, China; 3 Department of Ophthalmology, Shanghai Ninth People’s Hospital, Shanghai Jiao Tong University School of Medicine, 200011 Shanghai, China; 4 Department of Gastrointestinal Surgery, Renji Hospital, Shanghai Jiao Tong University School of Medicine, 200127 Shanghai, China

**Keywords:** Lung adenocarcinoma (LUAD), LAYN (Layilin), Immunotherapy, Tumor-infiltrating exhausted CD8^+^T, Anti-angiogenesis therapy

## Abstract

**Purpose:**

Our study intended to explore how low-dose anti-angiogenic drugs affected anti-tumor immunity of tumor-infiltrating exhausted CD8^+^T cells and achieved better clinical response when combined with immunotherapy. We set out to find potential targets or predictive biomarker on CD8^+^T cells for immunotherapy.

**Methods:**

We tested different doses of anti-VEGFR2 antibody combined with anti-PD1 antibody to treat LUAD in vivo and analyzed tumor-infiltrating CD8^+^T cells by flow cytometry. CD8^+^T cells overexpressing LAYN were co-cultured with LA795 cell lines to identify the function of LAYN in CD8^+^T cells. We also analyzed clinical samples from advanced LUAD patients treated with anti-angiogenesis therapy combined with immunotherapy.

**Results:**

Low-dose anti-VEGFR2 antibody combined with anti-PD1 antibody treatment delayed tumor growth and prolonged the survival time of tumor-bearing mice. The number of tumor-infiltrating CD8^+^T cells was reduced and the expression of LAYN was down-regulated in tumor-infiltrating CD8^+^T cells in the low-dose anti-VEGFR2 combination group. It was found that LAYN inhibited the killing function of CD8^+^T cells. In patients with advanced LUAD who received anti-angiogenesis therapy combined with immunotherapy, the LAYN^+^CD8^+^T cell subpopulation in good responders was significantly higher than that in poor responders. Furthermore, we demonstrated the expression of LAYN was regulated by upstream transcription factor NR4A1.

**Conclusion:**

Low-dose anti-VEGFR2 antibody combined with anti-PD1 antibody therapy promoted anti-tumor immunity and the downregulation of LAYN in tumor-infiltrating CD8^+^T cells played an important role in this process. These findings had implications for improving the efficacy of immune checkpoint blockade therapy and further optimized clinical treatment guidelines in advanced LUAD.

**Supplementary Information:**

The online version contains supplementary material available at 10.1007/s13402-022-00718-0.

## Introduction

Globally, lung cancer was still the leading cause of cancer morbidity and mortality [1]. Immune checkpoint blockade therapy had shown great promise in a variety of malignancies. However, though predictive biomarkers such as PD-L1 expression and tumor mutation burden could identify the patient subpopulation that responded to PD-1/PD-L1 mAbs, only 20% of NSCLC patients benefit from monotherapy [2, 3]. In order to extend the benefits to a larger population, combination with anti-angiogenesis therapy was increasingly applied to improve the response rate and efficacy of immunotherapy [4]. Studies had suggested the existence of a bidirectional relationship between angiogenesis and anti-tumor immunity in TME [5–7]. Combination of immunotherapy with anti-angiogenesis therapy improved the anti-tumor effect. Some pre-clinical studies had already observed promising anti-tumor effects in a variety of cancer types [8–11]. Exhausted CD8^+^T cells were the main cell type that responds to PD-1 blockade [12–15]. Exhausted CD8^+^T cell referred to a state of T cell dysfunction that occurred in chronic infections and cancers. It was defined as poor effector functions, continuous expression of inhibitory receptors, and transcriptional status different from functional effectors or memory T cells [16]. The tumor antigen-specific T cells in the tumor microenvironment were exhausted, and their anti-tumor function was greatly impaired [17, 18]. Exhausted T cells gradually lost the basic characteristics of effector T cells, such as effector cytokine production, cytotoxicity and proliferation [16, 19]. The modulating effect of anti-angiogenesis therapy on immunotherapy was related to dosage of anti-angiogenic drugs [20–23]. However, whether low-dose anti-angiogenesis therapy synergistically promoted anti-tumor immunity was lack of study. The original structure of the blood vessels in the solid tumor was broken, and blood perfusion was decreased, resulting in hypoxia in the tumor [24]. Hypoxia was a major regulator of vascular endothelial growth factor-A (VEGF-A) expression [25, 26]. Studies had shown that CD8^+^T cell exhaustion was driven by VEGF-A. VEGF-A induced the expression of the transcription factor TOX in T cells to drive the exhaustion specific transcription program in CD8^+^T cells [27]. In addition to promoting tumor blood vessel growth, VEGFA also had an immunosuppressive effect. The regulatory effects of anti-angiogenic drugs targeting VEGFA/VEGFR2 in immune cells, including CD8^+^T cells, Tregs, MDSCs, DCs, tumor-associated macrophages and mast cells [28] were worth of further study. Previous studies had found that LAYN was a key gene involved in tumor-infiltrating lymphocytes of liver cancer. Single-cell RNA sequencing of T cells confirmed that LAYN was up-regulated in exhausted CD8^+^T and Treg cells and inhibited the function of CD8^+^T cells in vitro [29]. Our study intended to identify how low-dose anti-angiogenic drugs affected the function of tumor-infiltrating CD8^+^T cells, and achieved better outcome by regulating expression of LAYN.

## Materials and methods

### Mice, tumor model and agents

6–8 weeks old, male C57BL/6 mice were purchased from Shanghai Jihui Experimental Animal Breeding Co., Ltd. (Shanghai, China). Animals were housed and maintained under optimal conditions of light, temperature, and humidity with free access to food and water. For subcutaneous tumor model, a total of 1 × 10^5^ LA795 cells were resuspended in 200 µL PBS and inoculated subcutaneously into the right flank of mice. Tumor dimensions were measured by caliper every other day, and tumor volume (mm^3^) was estimated using the formula: tumor volume = (length) × (width)^2^ × π/6. Different doses of anti-VEGFR2 antibody (DC101, BioXCell, BE0060) and IgG1 (BioXCell, BE0088) treatment were initiated 11 days after tumor cells inoculation and administered by intraperitoneal injection every 3 days. Anti-PD1 antibody (Anti-CD279, BioXCell, BE0146) and IgG2a (BioXCell, BE0089) treatment were initiated 12 days after tumor cells inoculation and was administered at 200 µg/mouse by intraperitoneal injection every 3 days. At indicated days later, the tumor-bearing mice were anesthetized and tissues were harvested for further analysis and measurement. Survival analysis was continued as independent experiments for indicated days.

### Cell lines

LA795 cells were purchased from Qingqi Biotechnology Development Co., Ltd. (Shanghai, China) and cultured at 37 °C in a humidified incubator with 5% CO2 in Roswell Park Memorial Institute (RPMI) 1640 Medium (Gibco) containing 10% fetal bovine serum (FBS) (Gibco) with 100 units/mL penicillin and 100 ug/mL streptomycin. The cells were routinely tested to confirm the absence of mycoplasma contamination and were cultured for a limited number of generations.

### Isolation of primary T cells from mouse models

Tumors from subcutaneous LA795 model were harvested on the days indicated. Tumor tissues were exhaustively flushed with PBS, minced into small pieces. Single cell suspensions were yielded through enzymatic digestion at 37°C for 40 min in RPMI 1640 medium containing collagenase type IV (1 mg/mL, Sigma) and DNase I (20 U/mL, Sigma). Triple volume FACS were added to stop the digestion. Filter the tissue digestion suspension into a new centrifuge tube with a 100 mesh filter, and centrifuge (350 g, 24 °C, 10 min). Add 40% Percoll to resuspend, use a 300 mesh filter into 15ml centrifuge tube, insert 3ml 80% Percoll into the bottom of the filtered cell suspension, and centrifuge (2500 rpm, 24 °C, 20 min). The lymphocyte layer was aspirated and transferred to a new 15ml centrifuge tube. FACS was added to the full volume, and centrifuged (400 g, RT, 10 min). Discard the supernatant and resuspend the pellet with FACS.

### Flow cytometry analysis

To determine intracellular cytokine expression, cells were stimulated with phorbol12-myristate 13-acetate (PMA), ionomycin, Golgistop for 4–6 h. At the end of stimulation, cells were stained with the indicated antibodies. Cells were stained in PBS containing 2% fetal bovine serum (FBS) with indicated antibodies for analysis of surface markers. For intracellular stain, cells were fixed in Fixation/Permeabilization according to the manufacturer’s instructions (eBioscience) for 40 min and then subjected to antibody staining. All samples were run on the BD LSRFortessa Flow Cytometer (BD Biosciences), and FlowJo software (TreeStar, Ashland, OR, USA) was used to analyze the data. Specific information on antibodies was listed in Table. S1. Tumor-infiltrating Immune cells were gated followed by Fig. S1.

### Immunohistochemistry and immunofluorescence

Paraffin-embedded samples were cut into 5-mm consecutive sections and deparaffinized. Specimens were incubated at 4 °C, overnight with antibodies against human LAYN, CD8, or against mouse CD8a, LAYN. Specimens were then washed by PBS and stained with anti-mouse/rabbit immunohistochemistry (IHC) secondary antibody kit. Double fluorescent staining with anti-CD8 and anti-LAYN was performed on snap-frozen sections embedded in OCT compound (8 mm), then 3–5 areas per tumor sample were randomly selected and evaluated by two pathologists who were blinded to patient information at 400×magnification. The results were determined based on both the staining intensity and the percentage of positive cells. The staining intensity was defined as follows: 0 = none; 1 = weak; 2 = intermediate; 3 = strong. The percentage of positive cells was scored as follows: 0–5%=0; 6–25%=1; 26–50%=2; 51–75%=3 and ≥ 76%=4. The final scores for the slides were determined by the two scores. A final score of < 4 was defined as low expression group, and ≥ 4 was marked as high expression group. Specific information on antibodies was listed in Table. S1.

### In vitro experiments

CD8^+^T cells isolated from human PBMC were cultured in the presence of plate-bound anti-CD3 (1 µg/ml) with different dosage VEGF-A (0, 20, 50ng/ml; R&D Systems). Cells were harvested and analyzed by flow cytometry after 84 h or used to extract mRNA after 60 h. T cells isolated from tumor of tumor-bearing mice were treated by the combination of antibody Anti-CD279 (BioXCell, BE0146) or IgG2a (BioXCell, BE0089) with different dosage anti-VEGFR2 antibody (DC101, BioXCell, BE0060) or IgG1 (BioXCell, BE0088). After 48 h, cells were analyzed by flow cytometry.

### Co-culture experiment

Mouse layilin was cloned into PLVX-IRES-PURO retroviral vector. Retrovirus was generated by using lentivirus packaging system before infecting OTI CD8^+^T cells. OTI CD8^+^T cells were isolated from the lymph nodes of OTI mice by using CD8a cell isolation kit (Miltenyi) and activated with OVA antigen peptide for 48 h. 48 h later, activated OTI CD8^+^T cells were spin-infected with retrovirus at 2000 rpm for 1 h under 32 °C. Puromycin was added to screen infection successful OTI CD8^+^T cells. OTI CD8^+^T cells with overexpression of LAYN were co-cultured with LA795-OVA for 48 h.After 48 h, killed LA795-OVA cells were detected by flow cytometry.

### Quantitative real-time polymerase chain reaction

Total RNA was extracted from cells according to the protocol of RNAprep Pure Micro Kit (TIANGEN; China). PrimeScript™ RT Reagent Kit (TaKaRa Bio; Shiga; Japan) was used to reverse transcribe mRNA into cDNA, and the SYBR Premix Ex Taq kit (Takara, RR420A) was used to quantitatively analyze the mRNA of the target gene. When testing, each sample was equipped with three replicate holes. β-Actin was used as an internal reference to calculate the relative expression of mRNA, and the formula was: RQ = 2−△△^Ct^. PCR primers were listed in Table. S2.

### Western blot

Samples lysed in RIPA buffer were cooked and then resolved by SDS-PAGE, followed by transferring to polyvinylidene fluoride membranes. Next, membranes were blocked with skimmed milk powder, incubated with the primary antibody overnight, and then incubated with the secondary antibody for 1 h. All experiments were replicated three times.

### Lentiviral transduction

To overexpressing LAYN on CD8^+^T cells, lentiviral transduction was performed by transfecting HEK293T cells with PHR-SFFV-EGFP lentiviral vectors encoding WT or LAYN along with lentivirus packaging system. To knock down NR4A1 on CD8^+^T cells, lentiviral transduction was performed by transfecting HEK293T cells with pLKO.1-GFP lentiviral vectors encoding WT or NR4A1 along with lentivirus packaging system. Isolated human CD8^+^T cells were activated with plate-bound anti-CD3 (1 mg/mL) and anti-CD28 (1 mg/mL) in 96-well plates for 72 h and then infected with the packaged lentivirus in the presence of 10 mg/ml polybrene by spinning at 2000 rpm for 1 h under 32 °C. CD8^+^T cells transduced with indicated lentiviral vectors were used for flow cytometric analysis or other experiments.

### Chip-qPCR

A total of 10^7^ CD8^+^T cells, isolated from human PBMC using CD8 isolation kit (Miltenyi Biotec), was activated with plate-bound anti-CD3 (1 ug/ml) and anti-CD28 (1 ug/ml) in 96-well plates for 72 h. Chromatin immunoprecipitation (ChIP) assays were performed according to the protocol of the SimpleChIP® Plus Sonication Chromatin IP Kit (56,383). NR4A1 antibody used for ChIP was purchased from Novus Biologicals. DNA isolated was tested by real-time quantitative PCR. The enrichment of samples in chromatin was based on the calculation formula, △CT = CTChIP DNA – CTInput DNA. Specific information on antibodies was listed in Table. S1.

### Patient samples

Written informed consent was obtained from all patients. Tumor samples were acquired from 8 patients with advanced lung adenocarcinoma (LUAD) who received pembrolizumab treatment (2 mg/kg body weight each time for every three weeks) combined with anlotinib treatment (12 mg, once daily for 2 weeks and stop the drug for 1 week) in the oncology department of Shanghai Changzheng Hospital. These patients were followed up from January 2014 to November 2021. The main selection criteria included patients with metastatic or unresectable recurrent lung adenocarcinoma (LUAD); measurable diseases in the Response Evaluation Criteria in Solid Tumors (RECIST 1.1); and had a representative tumor sample (paraffin). During the combination treatment, the tumor was evaluated every 8 weeks, and if the disease progresses, and we would change the treatment plan. Needle biopsy was used to collect tumor tissue before combination therapy. The analysis deadline for this study was November 2021.

### CRISPR-Cas9 targeting

The guide sequences against LAYN exons were designed and cloned into lenti-CRISPR v2 plasmid. CD8^+^T cells were transfected with lenti-CRISPR v2 plasmid according to the protocol of P3 Primary Cell 4D-Nucleofector™ X Kit. The primers of the guide RNAs targeting LAYN gene were as follows: Primer forward 1: CACCGGCCGTAGCGCCCGAGTGTCG. Primer reverse 1: AAACCGACACTCGGGCGCTACGGCC. Primer forward 2: CACCGCCGTAGCGCCCGAGTGTCGG. Primer reverse 2: AAACCCGACACTCGGGCGCTACGGC. Primer forward 3: CACCGCCGACACTCGGGCGCTACGG. Primer reverse 3: AAACCCGTAGCGCCCGAGTGTCGGC.

### Statistical analysis

Flow data were processed and analyzed using Flowjo10.0 software. GraphPad Prism software (version 8.0) was used for data statistical analysis and graphing. Data statistics were presented as mean ± standard (Mean ± SEM), and paired or unpaired T test (student t test) was used to test the significance of differences between two sets of data. The survival curve was drawn by the Kaplan-Meier method, and the difference was analyzed by the log-rank (Mantel-Cox) test. To compare the differences among the treatment groups in the in vivo study, one-way ANOVA was performed. P < 0.05 was considered statistically significant. In the figure, the symbols were used as: *, P < 0.05; **, P < 0.01; ***, P < 0.001; ****, P < 0.0001 by Student t test.

## Results

### LAYN was highly expressed in tumor-infiltrating exhausted CD8^+^T in patients with LUAD

**Fig. 1:**
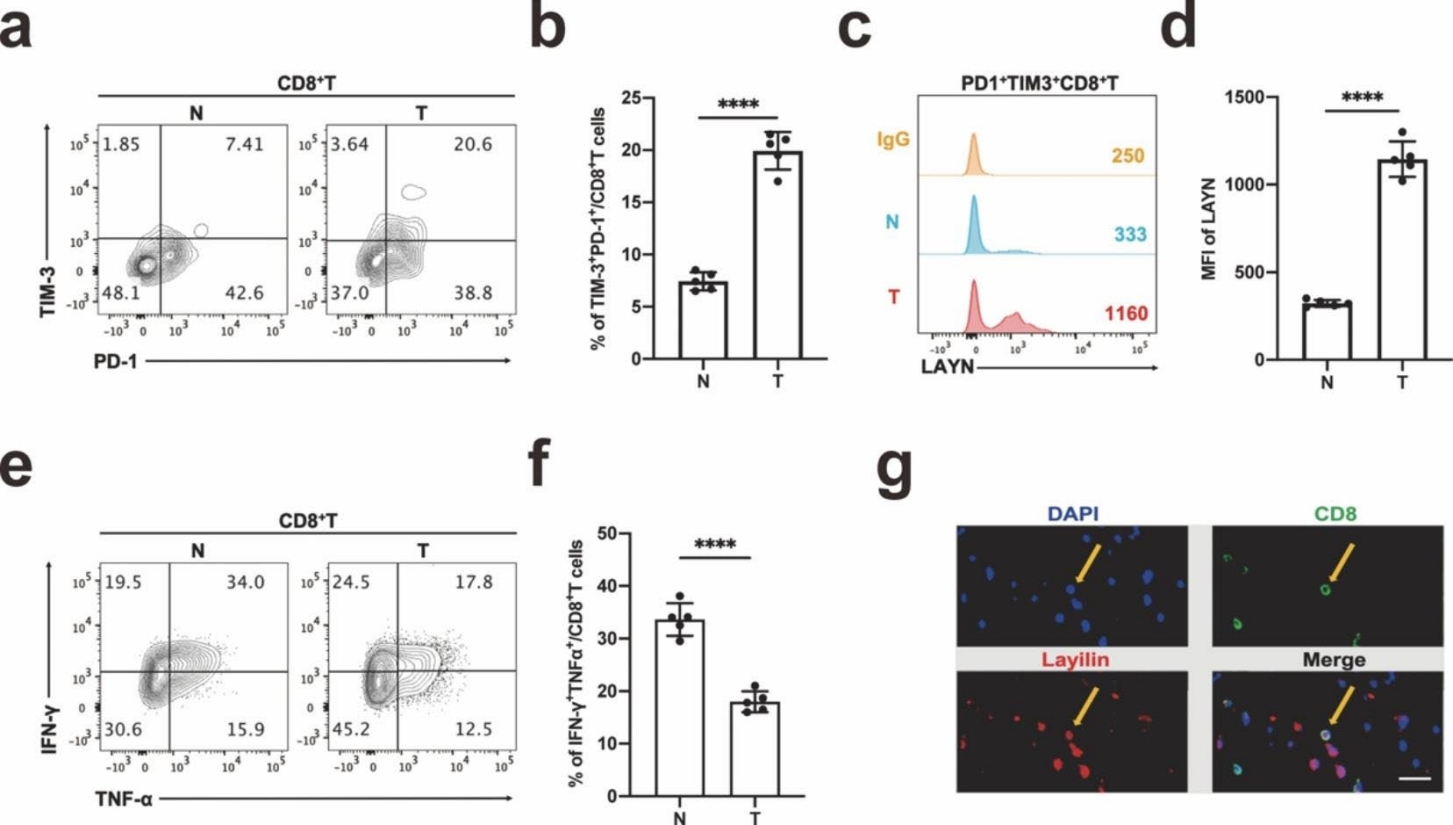
LAYN was highly expressed in lung cancer tumor-infiltrating exhausted CD8^**+**^**T (PD1**^**+**^**TIM3**^**+**^**CD8**^**+**^**T) cells.** (a, b) Percentage of exhausted CD8^+^T (PD1^+^TIM3^+^CD8^+^T) cells was detected in the tumor tissues and para-tumor tissues of patients with lung cancer by flow cytometry, and the results were performed by statistical analysis (n = 5 per group). (c, d) The expression of LAYN was detected on exhausted CD8^+^ T cells in tumor tissues and para-tumor tissues, and the MFI was performed by statistical analysis (n = 5 per group). (e, f) After PMA/Ionomycin and Golgi Stop stimulation, the ability of CD8^+^ T cells to secrete cytokines in para-tumor and tumor-infiltrating CD8^+^ T cells was tested, and the results were performed by statistical analysis (n = 5 per group). (g) Representative double immunofluorescence staining of CD8 and LAYN in the sections of resected lung cancer samples collected from patients was shown the location of LAYN (scale bar, 25 μm). Abbreviation: N, Normal (para-tumor tissues); T, Tumor (tumor tissues)

To detect the expression of LAYN in the tumor tissues of LUAD patients, we collected clinic samples and performed flow cytometry, immunohistochemistry, and immunofluorescence. The results showed that the proportion of tumor-infiltrating exhausted CD8^+^T (TIM3^+^PD1^+^CD8^+^T) cells was higher than that of para-tumor tissues by flow cytometry (Fig. 1a, b). At the same time, the MFI of LAYN on tumor-infiltrating exhausted CD8^+^T (TIM3^+^PD1^+^CD8^+^T) was significantly higher in tumor tissues than that in para-tumor tissues (Fig. 1c, d). In addition, the cytokines (TNF-α^+^IFN-γ^+^) secretion of CD8^+^T cells in the tumor tissues was less than that in para-tumor tissues, indicating that the function of CD8^+^T cells in tumor tissues was weaker than that in para-tumor tissues (Fig. 1e, f). The proliferation of tumor-infiltrating CD8^+^T cells was not statistically different from that of the para-tumor tissues (Fig. S2). The tumor tissues were embedded, dehydrated, and then subjected to immunofluorescence detection. It was found LAYN was co-located with CD8 in the membrane (Fig. 1 g). These data suggested that LAYN was highly expressed in tumor infiltrating CD8^+^T cells. The expression of LAYN on CD8^+^ T cells in non-exhausted environments such as peripheral blood was very low (Fig. S3).

### VEGF-A could up-regulate the expression of LAYN, NR4A1 and exhausted-related transcription factor TOX in CD8 + T cells

**Fig. 2:**
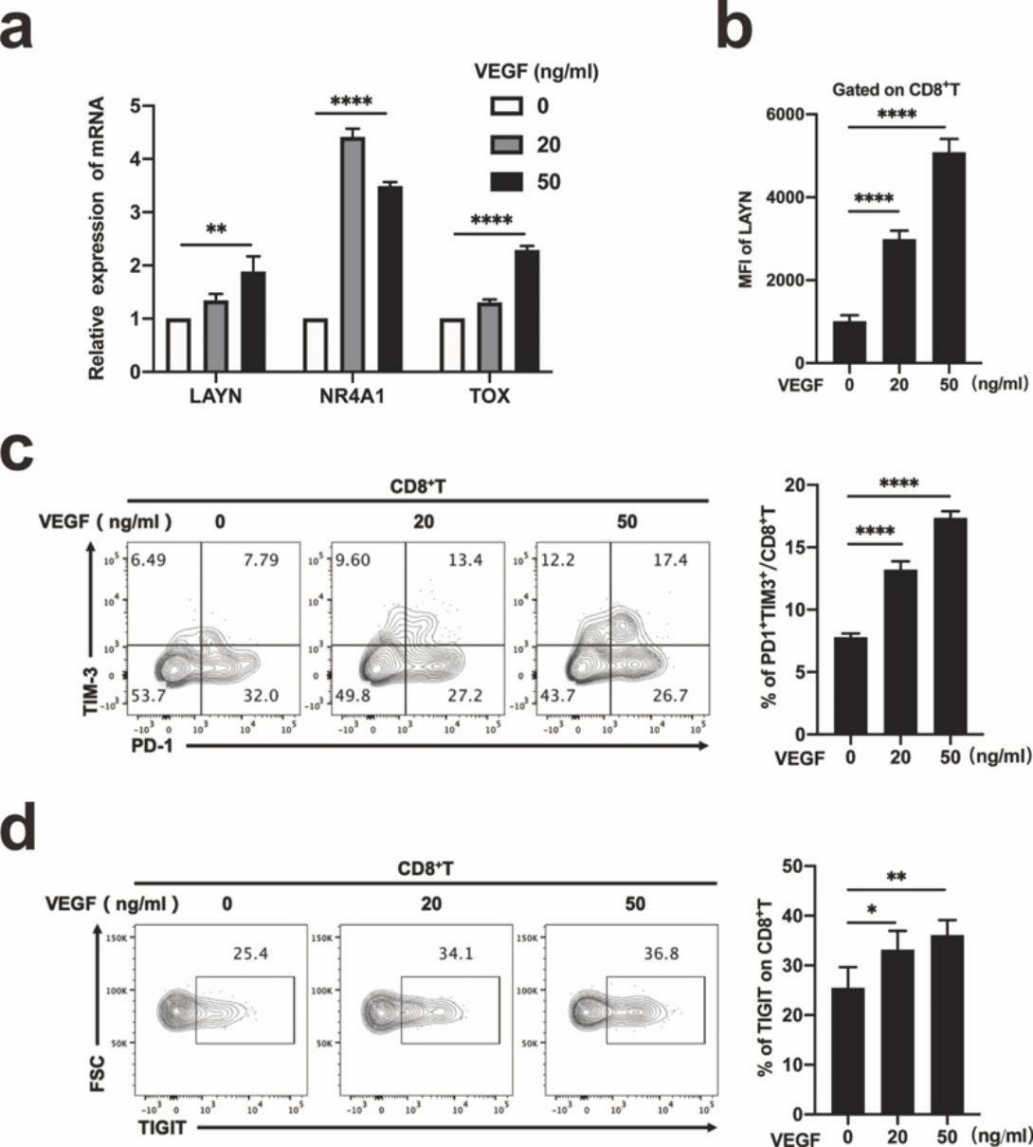
VEGF-A could up-regulate the expression of LAYN, NR4A1 and exhausted-related transcription factor TOX in CD^**+**^**T cells.** (a) CD8^+^ T cells from normal donors were activated by anti-CD3 antibody and treated with or without recombinant VEGF-A protein for 60 h. qPCR was performed to analyze the expression of LAYN, the exhaustion-related transcription factor TOX and NR4A1 (n = 3 per group). One representative experiment out of three was shown). (b) After 84 h, the MFI (mean fluorescence intensity) of LAYN on CD8^+^ T cells was detected by flow cytometry, and the results were performed by statistical analysis (n = 3 per group). One representative experiment out of three was shown. (c, d) After CD8^+^T cells treated with or without recombinant VEGFA protein for 84 h, the percentage of exhausted CD8^+^ T (PD1^+^ TIM3^+^ CD8^+^ T) cells was analyzed by flow cytometry (n = 3 per group). One representative experiment out of three was shown (c). The percentage of exhaustion-related marker TIGIT was also analyzed. Representative histograms were present (n = 3 per group). One representative experiment out of three was shown (d)

Next, we focused on identifying the driver responsible for regulating the expression of LAYN in the context of VEGF-A administration in CD8^+^T cell. In the absence of anti-CD3 antibody activation, the expression of LAYN, PD1, TIM3, and TIGIT on CD8^+^ T cells was low (Fig. S4a-d). After activation, the expression was up-regulated (Fig. S4a-d). The up-regulation of these immune checkpoints was a dual consequence of anti-CD3 antibody activation and VEGF-A treatment, but VEGF-A predominated. LAYN, NR4A1 and TOX were up-regulated genes after VEGF-A treatment in human CD8^+^T cells by qPCR (Fig. 2a). The up-regulation of LAYN proteins in human CD8^+^T cells treated by VEGF-A was analyzed by flow cytometry and showed by MFI (mean fluorescence intensity) (Fig. 2b). The percentage of PD1^+^TIM3^+^CD8^+^T(exhausted CD8^+^T) cells increased significantly after VEGF-A treatment in human CD8^+^T cells from donors (Fig. 2c). We confirmed that the VEGF-A induced up-regulation of not only LAYN but also immune checkpoint inhibitory receptor, such as TIGIT in human CD8^+^T cells (Fig. 2d). VEGF-A down-regulated the secretion of IFN-γ on activated CD8^+^ T cells gated from PBMC (Fig. S5a, b). VEGF-A could up-regulate the expression of LAYN and induced CD8^+^T cells exhaustion. The expression of VEGFR2 in tumor-infiltrating CD8^+^ T cells was significantly higher than that in normal tissues by flow cytometry (Fig. S6a, b). The expression of VEGFR2 on CD8^+^ T cells in PBMC was low, but the expression was significantly up-regulated after anti-CD3 antibody activation (Fig. S6c, d).

### The expression of LAYN was down-regulated in tumor-infiltrating exhausted CD8^**+**^T cells treated by the combination of low-dose DC101 with anti-PD1 antibody

**Fig. 3:**
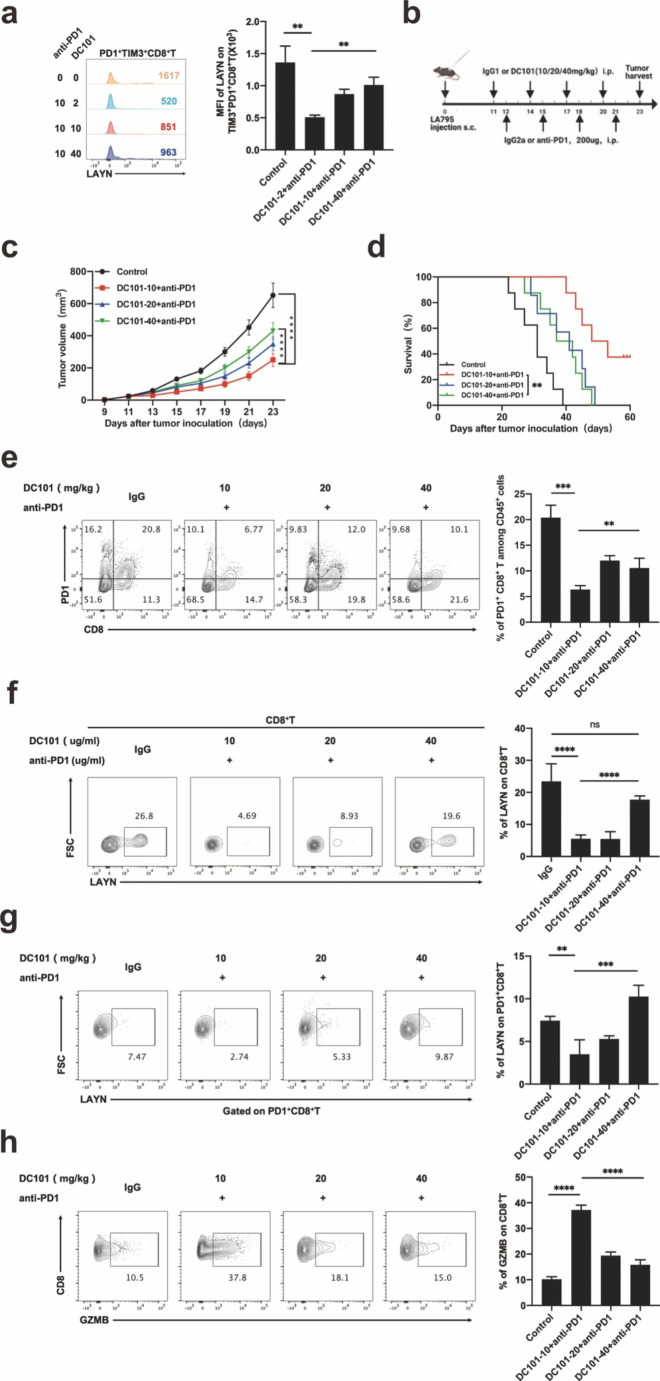
The expression of LAYN was down-regulated in tumor-infiltrating exhausted CD8^**+**^**T cells treated by the combination of low-dose DC101 with anti-PD1 antibody.** (a) For in vitro experiment, tumor-infiltrating CD8^+^ T cells enriched from tumor of tumor-bearing mice were cultured and treated for 48 h by combination of different dosages of DC101 with anti-PD1 antibody in vitro. The expression of LAYN was detected on exhausted CD8^+^T cells among different groups, and the MFI was performed by one-way ANOVA (n = 3 per group). One representative experiment out of three was shown. (b) For in vivo experiment, Mice were injected subcutaneously with LA795 cells to make a tumor-bearing mice model. They were given intraperitoneal injection of DC101 11 days after tumor cell inoculation and anti-PD1 antibody 12 days after tumor cell inoculation, DC101 and anti-PD-1 antibody were injected once every three days for a total of four times respectively. The tumor was harvested 23 days after tumor cell inoculation. (c) From 9 days after tumor cell inoculation, the tumor size was recorded every two days, and the tumor growth curve was performed by one-way ANOVA test (n = 5 per group). One representative experiment out of three was shown. (d) Survival of the LA795 tumor-bearing mice treated as indicated since the day of tumor cells injection was recorded and analyzed by log-rank (Mantel-Cox) test. (n = 8 per group). One representative experiment out of three was shown. (e, f, g, h) After all the administration of tumor-bearing mice was finished, analysis of tumor-infiltrating exhausted CD8^+^ T (PD1^+^ CD8^+^T) cells (e), LAYN on tumor-infiltrating CD8^+^T (LAYN^+^CD8^+^T) cells (f), LAYN on tumor-infiltrating exhausted CD8^+^T (LAYN^+^PD1^+^ CD8^+^T) cells (g) and GZMB^+^ CD8^+^ T cells (h) in LA795 tumors were performed by flow-cytometry and analyzed by one-way ANOVA (n = 5 per group). One representative experiment out of three was shown

To explore the relevant mechanism of low-dose anti-angiogenesis combined immunotherapy, we constructed lung adenocarcinoma tumor-bearing mice. Lymphocytes and tumor-infiltrating CD8^+^T cells were extracted from tumor tissues of tumor-bearing mice and treated with anti-PD1 antibody combined with different dosage anti-VEGFR2 antibody (DC101) in vitro. The results showed that in the low-dose anti-VEGFR2 antibody (DC101) group, the MFI of LAYN significantly decreased in tumor-infiltrating exhausted CD8^+^T cells (Fig. 3a), but the percentage of T cells treated by combination of different dosage of DC101 with anti-PD1 antibody in vitro didn’t change (Fig. S7). And then we performed in vivo experimental verification. Mice were injected subcutaneously with LA795 cell lines in the right flank. They were given intraperitoneal injection of DC101 11 days after tumor cell inoculation and anti-PD1 antibody 12 days after tumor cells inoculation. DC101 and anti-PD1 antibody were injected once every three days for a total of four times respectively. The tumors were harvested 23 days after tumor cell inoculation (Fig. 3b). It was found that low-dose anti-VEGFR2 antibody delayed tumor growth (Fig. 3c) and prolonged the survival time of tumor-bearing mice (Fig. 3d) compared with high-dose anti-VEGFR2 antibody. To further study its internal mechanism, we analyzed the expression and function of LAYN on tumor-infiltrating CD8^+^T cells in tumor tissues after treatment. The results showed that compared with the high-dose anti-VEGFR2 group, the percentage of tumor-infiltrating exhausted CD8^+^T (PD1^+^CD8^+^T) cells (Fig. 3e) and the expression of LAYN in the tumor-infiltrating CD8^+^T cells and tumor-infiltrating exhausted CD8^+^T (PD1^+^CD8^+^T) cells significantly were reduced in the low-dose anti-VEGFR2 group (Fig. 3f, g). At the same time, we found that GZMB secreted by tumor infiltrating CD8^+^T cells increased, indicating that the function of CD8^+^T cells were enhanced in the low-dose anti-VEGFR2 group (Fig. 3 h). However, there was no statistical difference in the percentage of T cells in the spleen among the groups (Fig. S8).

### LAYN downregulated the function of CD8^+^T cells

**Fig. 4:**
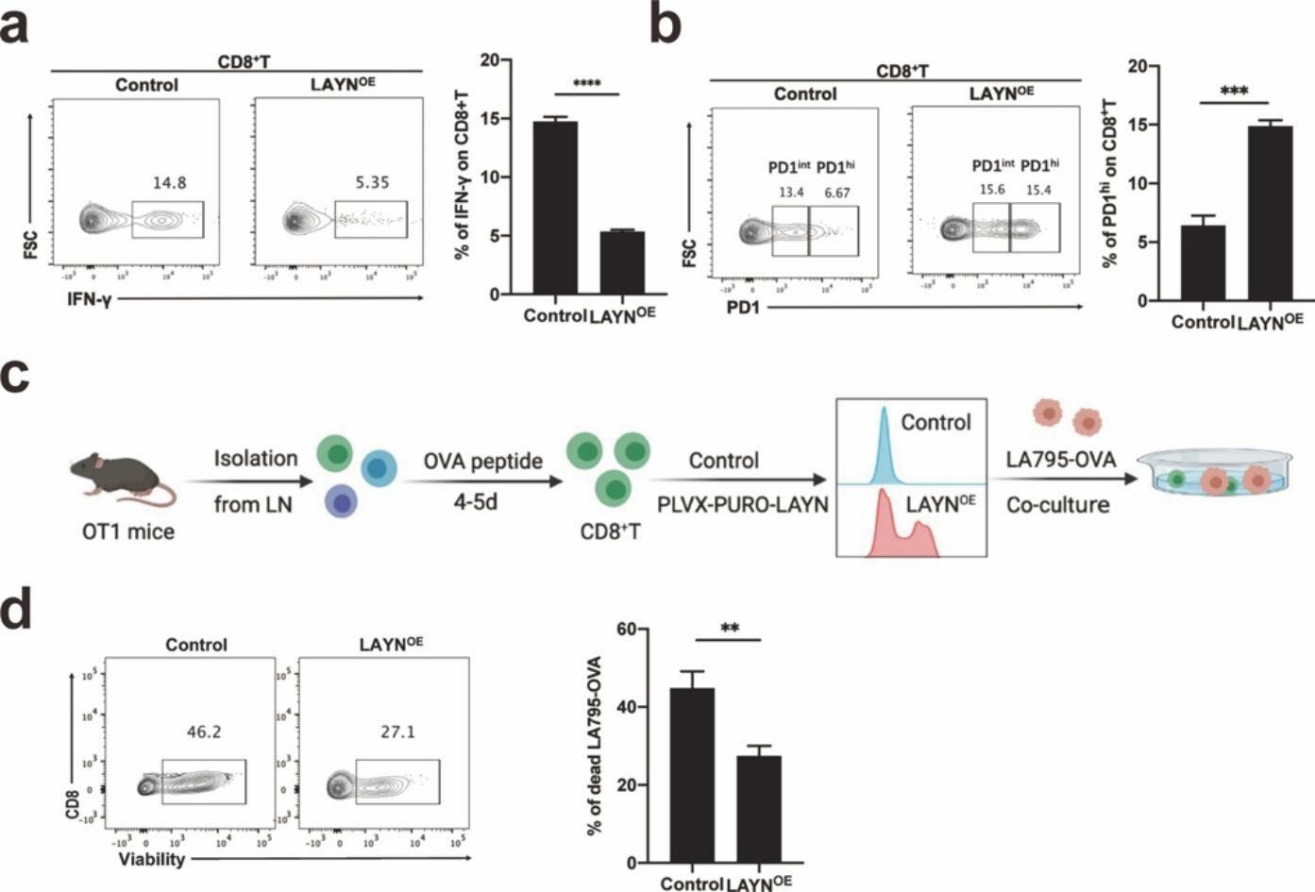
LAYN downregulated the function of CD8^**+**^**T cells.** (a) Analysis of cytokine IFN-γ secreted by CD8^+^ T cells overexpressing LAYN was performed by flow cytometry in vitro (n = 3 per group). One representative experiment out of three was shown. (b) Analysis of expression of PD1 on CD8^+^ T cells overexpressing LAYN was performed by flow cytometry in vitro (n = 3 per group). One representative experiment out of three was shown. (c) Lymphocytes isolated from lymph nodes of OTI mice were stimulated with OVA antigen peptide for 4–5 days and OTI CD8^+^T cells were isolated, infected with PLVX-PURO-LAYN/ PLVX-PURO-Vector plasmid for 48 h respectively. After that, OTI CD8^+^T cells overexpressing LAYN were co-cultured with LA795-OVA cells for 48 h. (d) LA795-OVA cells killed by OTI CD8^+^T cells were analyzed by flow cytometry (n = 3 per group). One representative experiment out of three was shown

To explore the role of LAYN in CD8^+^T cells, we identified its effect by overexpressing LAYN on CD8^+^T cells. The results showed that LAYN down-regulated the secretion of the cytokine IFN-γ (Fig. 4a), while up-regulating the expression of PD1 on CD8^+^T cells (Fig. 4b). Next, we conducted a specific killing experiment of OTI CD8^+^T cells against LA795-OVA cells in vitro. OTI CD8^+^T cells overexpressing LAYN were co-cultured with LA795-OVA (Fig. 4c). The results showed that the percentage of killed LA795-OVA cells by OTI CD8^+^T cells was significantly lower in OTI CD8^+^T cells overexpressing LAYN group than that in the control group by flow cytometry (Fig. 4d). The dead LA795-OVA cells killed by OTI CD8^+^T cells were gated (Fig. S9). Summary of these results: LAYN could inhibit the function of CD8^+^T cells. WT and CD8^+^T cells knocked out LAYN were treated with VEGFA, and the function of CD8^+^T cells in the knockout group was enhanced compared with that in the WT group (Fig. S10a, b). CD8^+^T cells overexpressed LAYN were treated with DC101 (anti-VEGFR2) or not respectively, the function of CD8^+^ T cells in DC101 treatment group was enhanced compared with that in untreated group (Fig. S10c, d). We proved that VEGF-A affected CD8^+^T cell function through regulating LAYN expression.

### The transcription factor NR4A1 bind to the promoter of LAYN and up-regulated the expression of LAYN

**Fig. 5:**
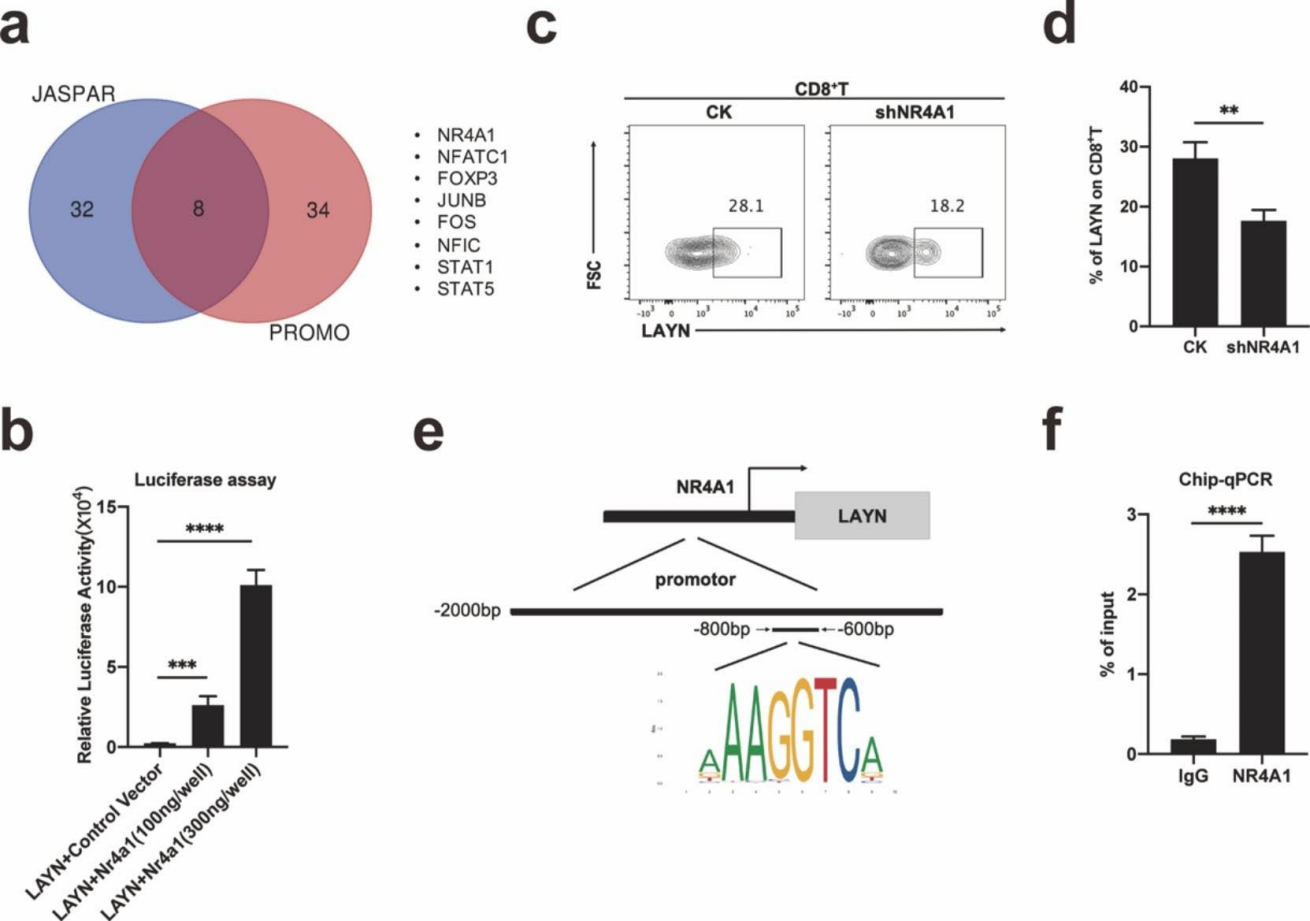
The transcription factor NR4A1 bind to the promoter of LAYN and up-regulated the expression of LAYN. (a) We predicted potential transcription factors of LAYN by PROMO and JASPAR websites, and then took the intersection. (b) Luciferase promoter assay was performed. 293T cells were transfected with the 2 kb promoter region of LAYN and the empty vector or the NR4A1 overexpression vector at different concentrations. About 48 h after transfection, the luciferase activity was measured (n = 3 per group). One representative experiment out of three was shown. (c, d) By knocking down NR4A1 in CD8^+^T cells, the percentage of LAYN expression was analyzed by flow cytometry (n = 3 per group). One representative experiment out of three was shown. (e, f) The Chip experiment was performed by using the antibody of NR4A1, qPCR was used to measure the relative enrichment of the LAYN promoter in the Chip experiment, Chip-qPCR confirmed the binding of the predicted TF (Transcription factor) at the locations of the enhancer site (n = 3 per group). One representative experiment out of three was shown

To find the upstream regulating mechanism of LAYN in CD8^+^T cells, we predicted the potential transcription factors regulating LAYN on PROMO and JASPAR websites. The intersection of the two databases contained 8 transcription factors (Fig. 5a). Through the Luciferase experiment, we found that NR4A1 up-regulated the expression of LAYN at the transcription level (Fig. 5b). After knocking down NR4A1 on CD8^+^T cells by shRNA, the expression of LAYN on CD8^+^T cells was down-regulated by flow cytometry analysis (Fig. 5c, d). We conducted a Chip-qPCR experiment to further find the region of NR4A1 binding to the promoter of LAYN. It was found that the region where NR4A1 bounded to the promoter of LAYN ranged from − 800 bp to -600 bp (Fig. 5e). Statistical analysis of Chip-qPCR results showed significant differences (Fig. 5f).

### The expression of LAYN on tumor-infiltrating CD8^**+**^**T cells was correlated with LUAD patients’ response to the combination of anti-angiogenesis therapy with immunotherapy**

**Fig. 6:**
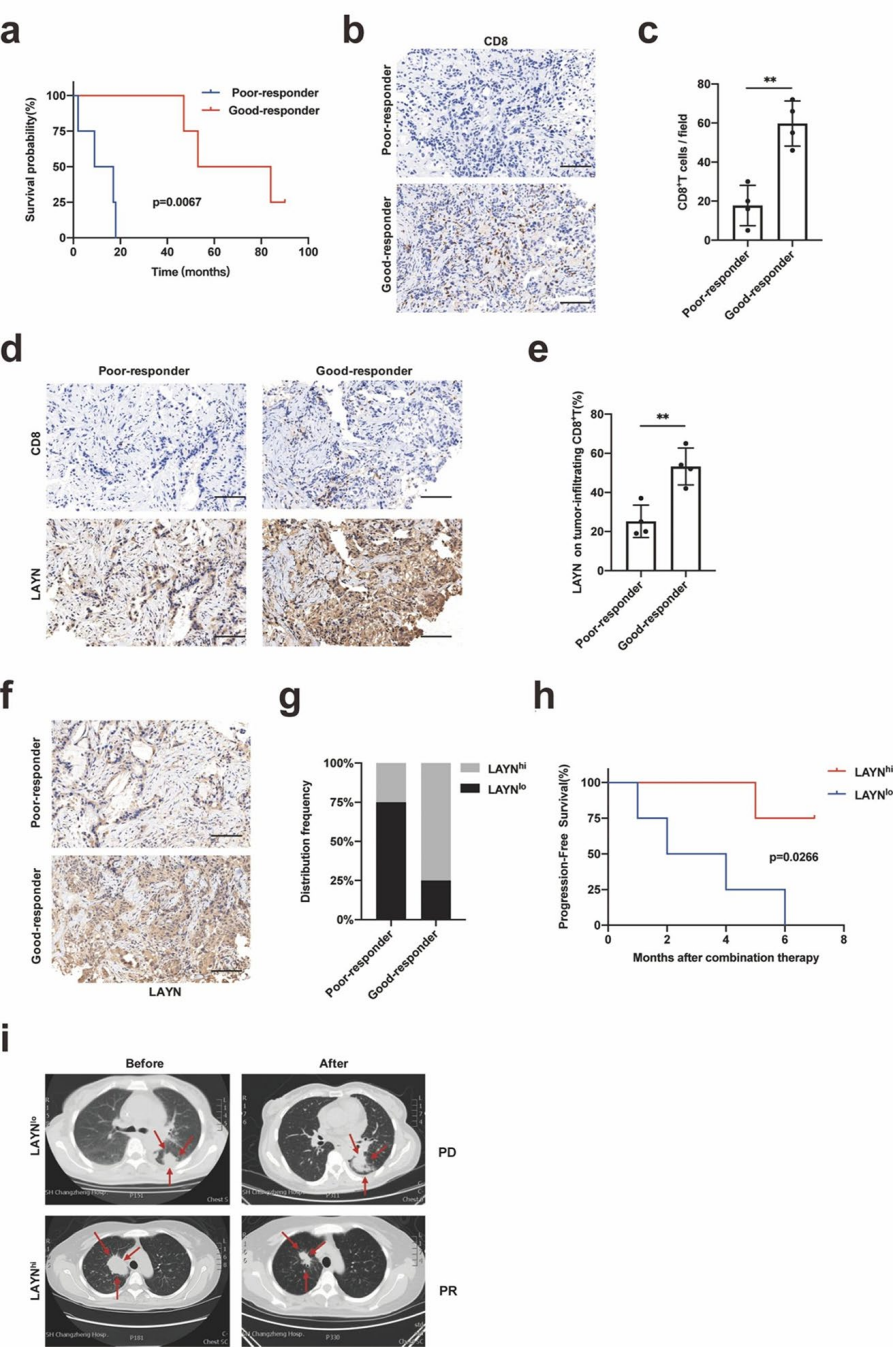
The expression of LAYN on tumor-infiltrating CD8^**+**^**T cells was correlated with LUAD patients’ response to the combination of anti-angiogenesis therapy with immunotherapy.** (a) The Kaplan–Meier curves for OS of advanced LUAD patients in the good-responder and poor-responder groups who received anti-angiogenesis therapy combined with immunotherapy (n = 4 per group). (b, c) Representative immunohistochemistry (IHC) staining of CD8 (scale bar, 100 μm) in advanced lung adenocarcinoma samples collected from good-responder and poor-responder patients before combination therapy. Each dot indicated one tumor per patient and represented the average of 5–8 images (n = 4 per group). (d, e) Representative immunohistochemistry (IHC) staining of CD8 and LAYN (scale bar 100 μm) in advanced LUAD samples collected from good-responder and poor-responder patients before combination of anti-angiogenesis therapy with immunotherapy. Each dot indicated one tumor per patient (n = 4 per group). (f, g) Representative immunohistochemistry (IHC) staining of LAYN on CD8^+^ T cells in advanced lung adenocarcinoma (LUAD) samples collected from good-responder and poor-responder patients before combination of anti-angiogenesis therapy with immunotherapy. Good-responders and poor-responders showed frequencies of patients with high and low expression of LAYN in tumor. The cutoff final scores for high and low expression of LAYN were ≥ 4 and < 4 respectively. (h) The Kaplan–Meier curves for PFS (Progression-Free Survival) of advanced LUAD patients in the LAYN^hi^ and LAYN^lo^ groups who received combination of anti-angiogenesis therapy with immunotherapy (n = 4 per group). (i) Tumor CT scans of the patients in the LAYN^hi^ and LAYN^lo^ groups were performed. A representative patient from each group was shown respectively, the red arrows denoted the target lesions change after combination of anti-angiogenesis therapy with immunotherapy. Representative patient of good-responder (PR) in LAYN^hi^ group and poor-responder (PD) in LAYN^lo^ group was shown respectively. Abbreviation: PR, Partial Remission; PD, Progression Disease

Samples of tumor tissues were collected before combination therapy. At the cutoff date for data analysis of this study, a total of 8 patients were enrolled and received anlotinib combined with pembrolizumab. We stratified the patients into good-responder and poor-responder groups based on their survival time(p = 0.0114) (Fig. 6a). By immunohistochemical analysis of tumor tissues obtained before combination therapy, we performed immune profiling of the tumor-infiltrated cells and found that good-responders had more tumor-infiltrating CD8^+^T cells than poor-responders (Fig. 6b, c). The percentage of LAYN expression on tumor-infiltrating CD8^+^T cells was higher in good-responder group than those in poor-responder group before combination therapy (Fig. 6d, e). We also performed double immunofluorescence staining of LAYN and CD8 on tumor tissue samples (Fig. S11a, b). According to immunohistochemical analysis of tumor tissues obtained before combination therapy, we found the patients with high expression of LAYN (LAYN^hi^) in the good-responders group accounted for 75%, low expression of LAYN (LAYN^lo^) in the good-responders group accounted for 25%, in contrast, 25% patients with high expression of LAYN (LAYN^hi^) and 75% patients with low expression of LAYN (LAYN^lo^) were observed in the poor-responders group (Fig. 6f, g). PFS (Progression-Free Survival) of LAYN^hi^ group was significantly longer than that of LAYN^lo^ group (p = 0.0266) (Fig. 6 h). According to the RECIST criteria (version 1.1), the primary lung lesions of all the eight patients before and after combination treatment were measured, and it was easy to achieve PR (Partial Remission) in the LAYN^hi^ patient group, but PD (Progression Disease) in the LAYN^lo^ patient group, (Fig. 6i).

## Discussion

Lung cancer was the leading cause of cancer-related death, and many patients with lung cancer were already in the advanced stage at the time of diagnosis. Anti-angiogenesis therapy and blockade of immune checkpoint therapy (immunotherapy) had been widely used in the treatment of lung cancer in recent years due to their high efficiency and low toxicity. Clinical studies had reported that the combined application of anti-angiogenesis therapy and immunotherapy could produce a strong synergistic effect in both first-line and subsequent-line treatments. Also, low-dose anti-angiogenic therapy combined with immunotherapy could produce stronger anti-tumor effects than high-dose anti-angiogenic therapy combined with immunotherapy, but the specific mechanism was still unknown.

We constructed mice model by subcutaneously injected LA795 cells, and then administered different doses of DC101 combined with anti-PD-1 in mice by intraperitoneal injection. Dual-drug combination therapy was a current trend and one of the hottest research fields. The combination of various drugs and anti-PD-1/PD-L1 immunotherapy was a topic worth of studying [30–32]. Some advanced NSCLC patients had long-lasting clinical benefits for single-drug therapy, but there were still a considerable number of patients who couldn’t benefit from monotherapy at all [33]. Based on previous research reports and our own research, we had found that low-dose anti-angiogenesis combined immunotherapy could achieve better anti-tumor effects, and the expression of LAYN on tumor-infiltrating CD8^+^T cells was negatively correlated with the prognosis of patients with lung adenocarcinoma.

According to our experimental results, VEGF-A induced the up-regulation of LAYN, NR4A1 and TOX on CD8^+^T cells, and the expression of immunosuppressive receptors (TIGIT, LAG3, TIM3, PD-1) also increased. After activation of CD8^+^T cells, a large amount of VEGF-A was produced [34]. Previous related studies had established five tumor models and found that anti-VEGF-A treatment improved the production of effector cytokines of tumor-infiltrating CD8^+^T cells. Similarly, after treatment with anti-VEGFR2 or anti-angiogenin 2 and VEGF-A bispecific antibodies, the disruption of VEGFR2 signaling enhanced the effector function of T cells in ovarian cancer patients and mouse tumors [35–38] which was consistent with our results. Anti-VEGF-A strengthened the activation of CD8^+^T cells in tumors and enhanced their ability to produce cytokines by initiating the hypoxia-inducible factor-1 (HIF-1⍺) program in CD8^+^T cells to directly promote the production of cytokines [39]. This was different from our research results and the research of Gavalas NG et al. The components of the tumor microenvironment were relatively complex, and different tumor types and malignant burdens might cause different research results.

Our research results showed that anti-PD-1 combined with low-dose anti-VEGFR2 therapy significantly delayed tumor growth and prolonged the survival of mice in preclinical models of LUAD, revealing that anti-VEGFR2 therapy depended on different doses to affect immunotherapy. The effect of anti-angiogenic drugs on the immune microenvironment was dose-dependent [8]. In many types of cancer, relevant studies had confirmed that low-dose anti-angiogenic drugs promoted anti-tumor immunity [22, 23, 40]. But the mechanism was currently lack of studies.

As mentioned above, exhausted CD8^+^T cells did not completely lose its function. Under certain conditions, their vitality could be restored to enhance anti-tumor immunity. Therefore, certain differential genes expressed in tumor-infiltrating CD8^+^T cells of low-dose anti-angiogenesis combined immunotherapy group might be potentially responsible for the functional recovery of tumor-infiltrating exhausted CD8^+^T cells.

To further explore the mechanism of low-dose anti-angiogenic drugs promoting anti-tumor immunity. Our research found that compared with high-dose anti-VEGFR2 combined with anti-PD-1, low-dose anti-VEGFR2 combined with anti-PD-1 down-regulated the expression of LAYN on tumor infiltrating exhausted CD8^+^T cells, increased the secretion of GZMB, and then enhanced the function of CD8^+^T cells. Our results showed that LAYN was a key factor of regulation.

Layilin was a 55 kDa transmembrane protein homologous to C-type lectin and was expressed in many cell types and organs. Layilin acted as a surface receptor for hyaluronic acid (HA). Therefore, LAYN played an important role in cell adhesion, proliferation and migration [41, 42]. Low level of LAYN protein reduced cell invasion and lymph node metastasis in A549 lung cancer cells, which was the first proof that LAYN was related to cancer [43]. LAYN was also involved in the epithelial-mesenchymal transition (EMT) of renal tubular epithelial cells induced by tumor necrosis factor-α (TNF-α) [44], and played a key role in the tight junctions of the intestinal epithelium induced by HA35 in inflammatory bowel disease [45]. There were research data showing that the high expression of LAYN was related to the poor prognosis of patients with CRC and NSCLC [46], the studies of LAYN were mostly in parenchymal cells rather than mesenchymal cells. These findings indicated that LAYN played an important role in cancer progression, invasion, and metastasis, and most of these studies were based on tumor cells. However, there were few studies on LAYN in immune cells.

In recent years, LAYN had begun to be studied on T cells and gradually been reported in bioinformatic studies. Relevant studies had shown that LAYN was also expressed in TILs isolated from several human cancers [15, 46, 47]. However, the research on the function of immune cells by this molecule was limited. There were few studies on the function of LAYN in lymphocytes reported so far. One of the studies showed that LAYN reduced the ability of CD8^+^T cells to secrete IFN-γ25, which was consistent with our findings. Another study showed that LAYN (Layilin) promoted the activation of integrin to enhance anti-tumor immunity, which was completely contrary to our findings [48]. We designed experiments by overexpressing LAYN on CD8^+^T cells in LUAD, but Mahuron KM designed experiments by knocking out LAYN on CD8^+^T cells in melanoma. The difference of research results might be caused by the different experimental design and cancer types. All of these were worth of our further study. Did LAYN promote immunosuppression like immune checkpoint such as PD1? Our research found that LAYN indeed down-regulated the expression of cytokines in CD8^+^T cells and affected the killing function of CD8^+^T cells. At the same time, we carried out tumor-specific killing experiments in vitro and obtained such results.

How VEGFA/VEGFR2 regulated the expression of LAYN on CD8^+^T cells remained to be further studied. We found that VEGF-A up-regulated NR4A1 and LAYN, and the transcription factor NR4A1 positively regulated LAYN. However, whether the up-regulation of LAYN by VEGFA depended on NR4A1 still needed to be studied in depth. This study provided a new theoretical basis for the clinical application of anti-PD-1 combination therapy strategies. LAYN might become a potential target for cancer treatment.

To further find the upstream regulation of LAYN, we predicted the relevant transcription factors that were up-regulated after VEGFA-treated CD8^+^T cells from normal donors. We identified the transcription factor NR4A1, which was confirmed by Luciferase and Chip-qPCR experiments. At the same time, how the activation of the downstream of LAYN affected other signal pathways, influencing the secretion of cytokines (such as IFN-γ, GZMB), and many other mechanisms that regulated anti-tumor immunity were worth of further investigation.

In conclusion, LAYN was highly expressed on tumor-infiltrating CD8^+^T cells in tumor tissues of patients with LUAD. The expression of LAYN was positively correlated with the LUAD patient’s response to the combination of anti-angiogenesis therapy with immunotherapy. Low-dose anti-VEGFR2 combined with anti-PD-1 therapy promoted anti-tumor immunity, and its mechanism was related to the down-regulation of LAYN on tumor-infiltrating CD8^+^T cells. LAYN might be a potential target or a biomarker of patient response to tumor immunotherapy.

## Electronic supplementary material

Below is the link to the electronic supplementary material.

Supplementary Material 1

## Data Availability

Data related to this study can be obtained from the corresponding author.

## Abbreviations

LUAD: Lung adenocarcinoma
PD-1: Programmed cell death protein-1
IFN-γ: Interferon-γ
TNF-α: Tumor necrosis factor-α
