# Supplementary material for: Low-dose anti-VEGFR2 therapy promotes anti-tumor immunity in lung adenocarcinoma by down-regulating the expression of layilin on tumor-infiltrating CD8+T cells
Source: Cell Oncol (Dordr). 2022 Oct 19;45(6):1297–309. doi: 10.1007/s13402-022-00718-0 (PMC9747853; doi:10.1007/s13402-022-00718-0)
Supplement: Supplementary file 1 — Supplementary Material 1 [file 13402_2022_718_MOESM1_ESM.pdf]

Table. S1

Name	Company	Catalog
Layilin/LAYN Antibody, RabbitPAb	Sinobiological	80431-RP01
Layilin/LAYN Antibody, MouseMAb	Sinobiological	10208-MM02
Alexa Fluor™ 647 Antibody Labeling Kit	Invitrogen™	A20186
Anti-human CD279 (PD-1) BV421	BD Pharmingen	564323
Anti-human TIGIT BV786	BD Pharmingen	747838
Anti-human CD4 BB515	BD Pharmingen	564419
Anti-human CD8 BB700	BD Pharmingen	566452
Brilliant Violet 421™ anti-mouse CD8a	Biolegend	100753
PE anti-human/mouse Granzyme B Recombinant	Biolegend	372208
Layilin Polyclonal Antibody	Thermo Fisher	PA5-59061

Table. S1 Specific information on antibodies of Immunohistochemistry, immunofluorescence, and flow cytometry analysis

Table. S2

Primer	sequence
hβ-ACTIN-F	CTCTTCCAGCCTTCCTTCCT
hβ-ACTIN-R	CAGGGCAGTGATCTCCTTCT
hTOX-F	TATGAGCATGACAGAGCCGAG
hTOX-R	GGAAGGAGGAGTAATTGGTGGA
hLAYN-F	GCGTGGTCATGTACCATCAG
hLAYN-R	AGGTGTTGTCAGCTCTGTTTC
hNR4A1-F	CCCTGAAGTTGTTCCCCTCAC
hNR4A1-R	GCCCTCAAGGTGTGGAGAAG

Table. S2 Primers for PCR

Fig. S1

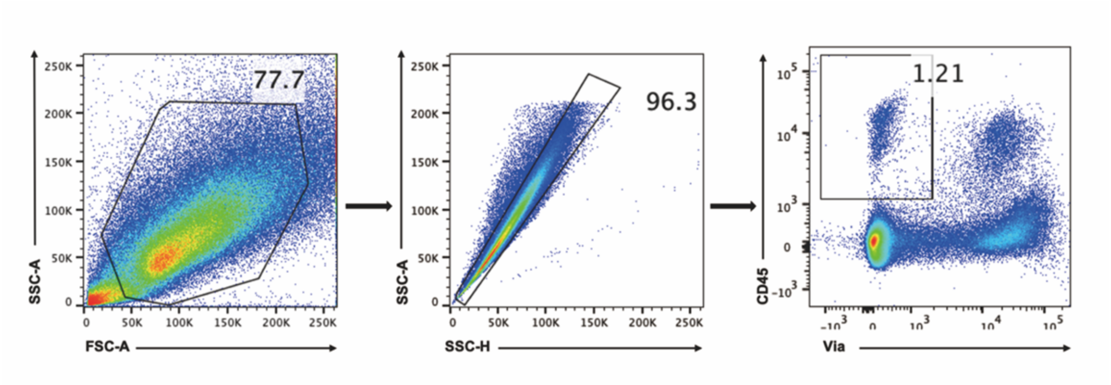

Fig. S1 Tumor-infiltrating Immune cells Gating

Fig. S2

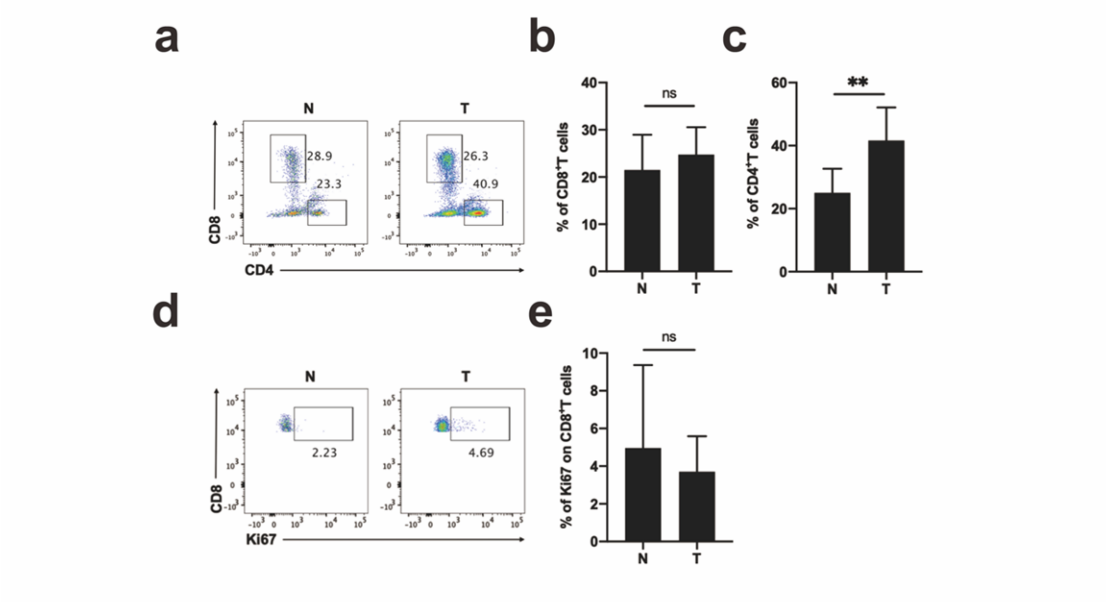

Fig. S2 The proliferation of tumor-infiltrating CD8⁺T cells was not statistically different from that of the para-tumor tissues. (a, b, c) The percentage of CD8⁺T and CD4⁺T cells was detected in the tumor tissues and para-tumor tissues of patients with lung cancer by flow cytometry, and the results were performed by statistical analysis (n=5). (d, e) The proliferation of CD8⁺T cells was detected in tumor tissues and para-tumor tissues, and the results were performed by statistical analysis (n=5 per group). One representative experiment out of three was shown. Abbreviation: N, Normal (para-tumor tissues); T, Tumor (tumor tissues).

Fig. S3

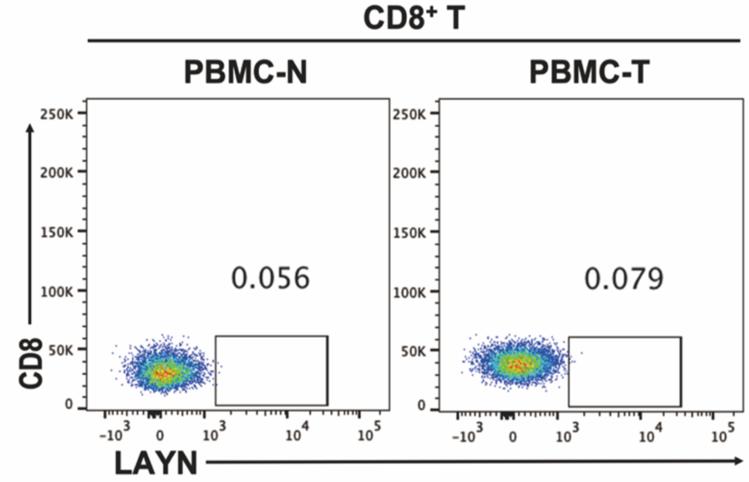

Fig. S3 LAYN on CD8⁺ T cells in non-exhausted environments such as PBMC was hardly expressed. The expression of LAYN on CD8⁺ T cells from normal donors' PBMC (PBMC-N) and tumor patients' PBMC (PBMC-T) was analyzed by flow cytometry (n=4 per group).

Fig. S4

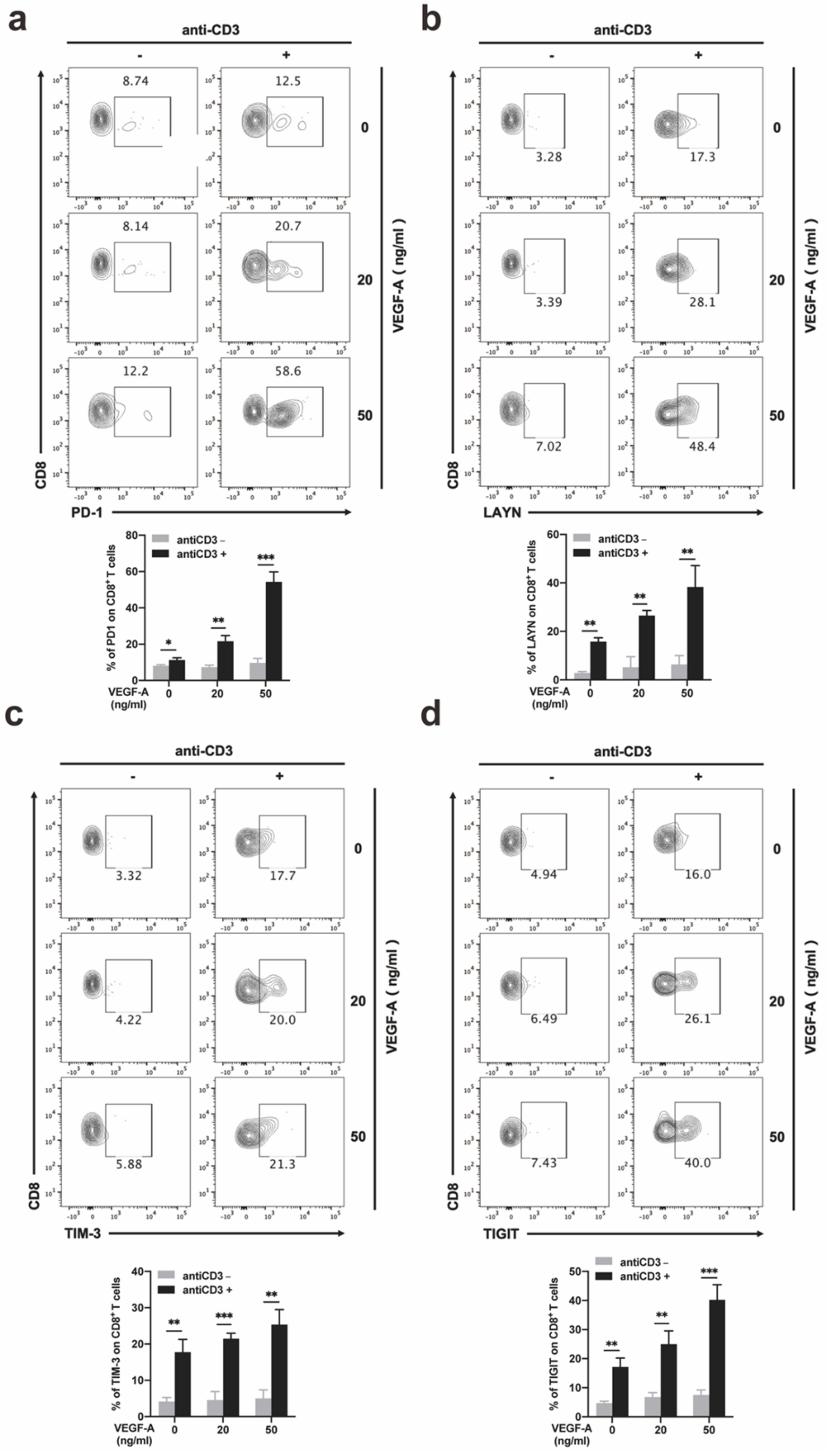
a
anti-CD3
-
+
8.74
12.5
8.14
20.7
12.2
58.6
0
20
50
VEGF-A (ng/ml)
CD8
PD-1
antiCD3 –
antiCD3 +
% of PD1 on CD8⁺ T cells
VEGF-A (ng/ml)
b
anti-CD3
3.28
17.3
3.39
28.1
7.02
48.4
LAYN
% of LAYN on CD8⁺ T cells
c
anti-CD3
3.32
17.7
4.22
20.0
5.88
21.3
TIM-3
% of TIM-3 on CD8⁺ T cells
d
anti-CD3
4.94
16.0
6.49
26.1
7.43
40.0
TIGIT
% of TIGIT on CD8⁺ T cells

Fig. S4 The basal level of LAYN, PD1, TIM3 or TIGIT without anti-CD3 stimulation was low. (a-d) With anti-CD3 antibody activation or in the absence of anti-CD3 antibody activation, the expression of LAYN, PD1, TIM3, and TIGIT on $CD8^+$ T cells in PBMC was analyzed by flow cytometry after treatment with different doses of VEGF-A (n=3 per group). One representative experiment out of three was shown.

Fig. S5

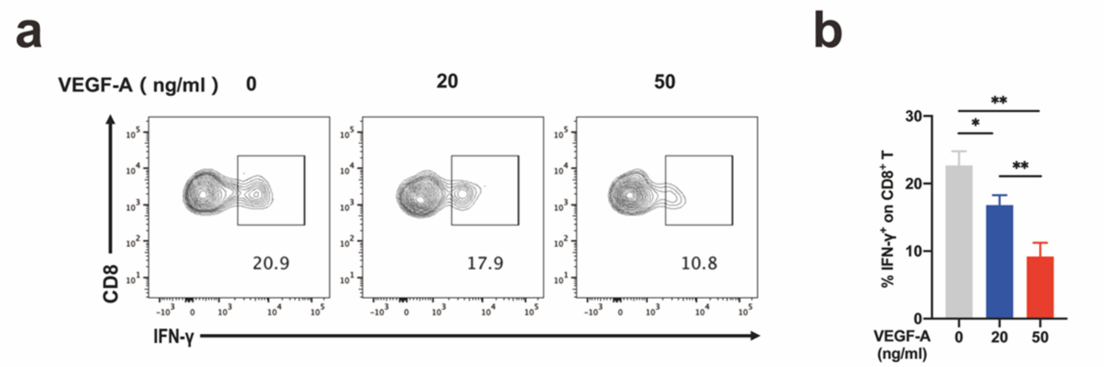

Fig. S5 VEGF-A down-regulated the secretion of IFN-γ on activated CD8$^+$ T cells gated from PBMC. (a, b) With anti-CD3 antibody activation, the expression of IFN-γ on CD8$^+$ T cells in PBMC was analyzed by flow cytometry after treatment with different doses of VEGF-A (n=3 per group). One representative experiment out of three was shown.

Fig. S6

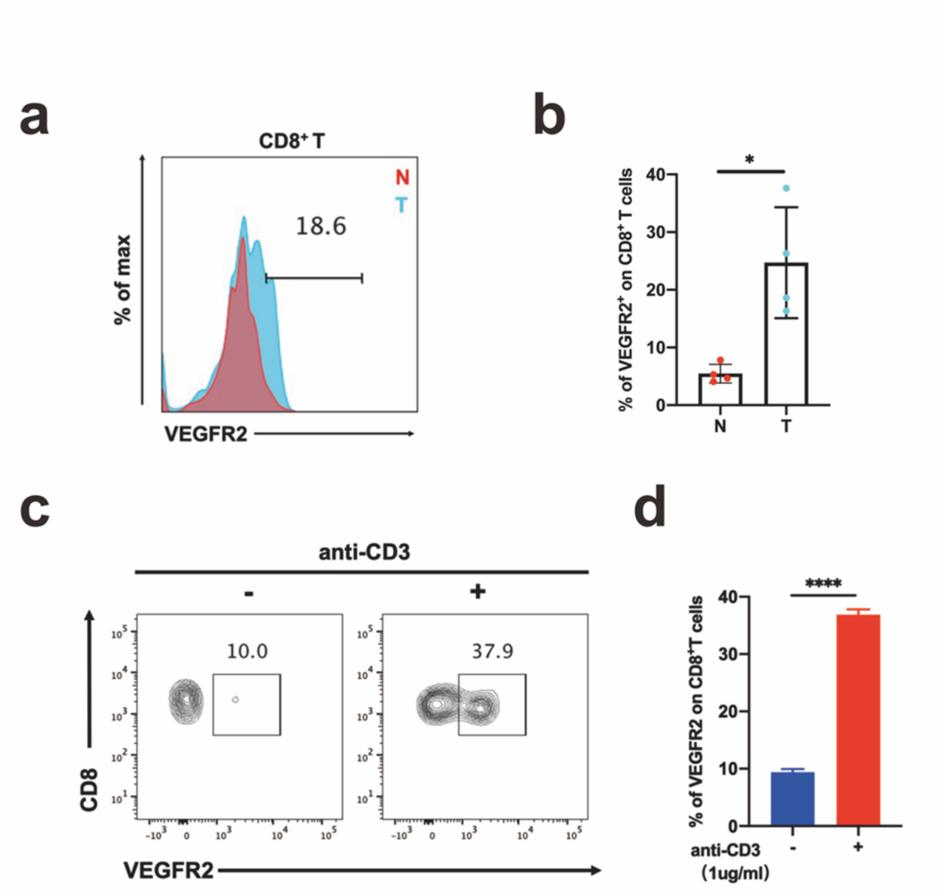

Fig. S6 The expression of VEGFR2 in tumor-infiltrating CD8$^+$ T cells was significantly higher than that in normal tissues. (a, b) The expression of VEGFR2 on CD8$^+$ T cells from normal tissues (N) and tumor tissues (T) was analyzed by flow cytometry (n=4 per group). Representative histograms were present. (c, d) CD8$^+$ T cells were obtained from normal donors and stimulated with anti-CD3 antibodies for 84 hours. The expression of VEGFR2 was analyzed by flow cytometry (n=3 per group). Representative histograms were present.

Fig. S7

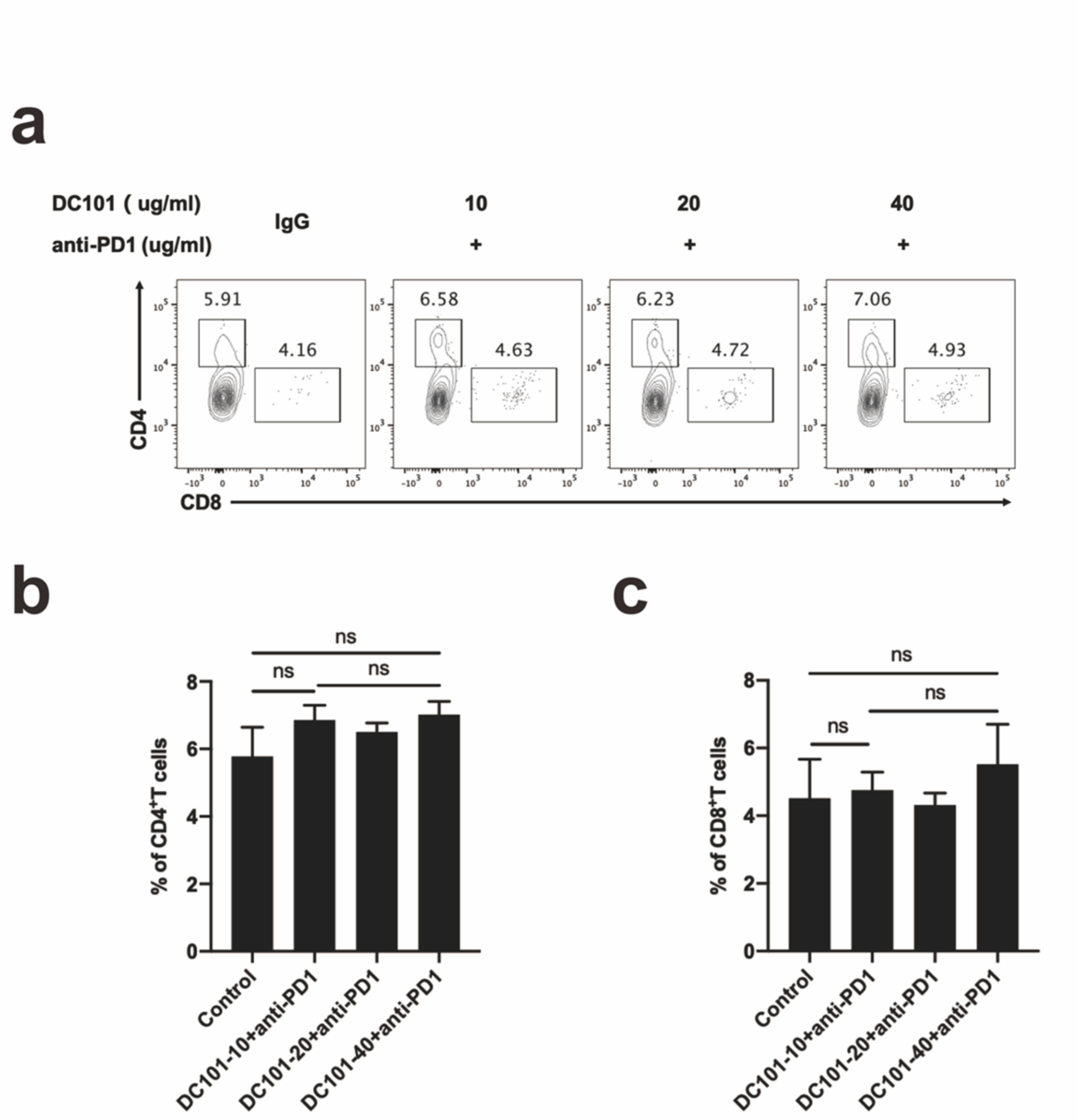

Fig. S7 Percentage of T cells enriched from tumor of tumor-bearing mice treated by combination of different dosage of DC101 with anti-PD1 antibody in vitro didn't change. (a, b, c) $CD8^{+}$T cells enriched from tumors of tumor-bearing mice were treated by combination of different dosages of DC101 with anti-PD1 antibody in vitro. The percentage of $CD8^{+}$T and $CD4^{+}$T cells was detected by flow cytometry, and the results were performed by statistical analysis (n=3 per group). One representative experiment out of three was shown.

Fig. S8

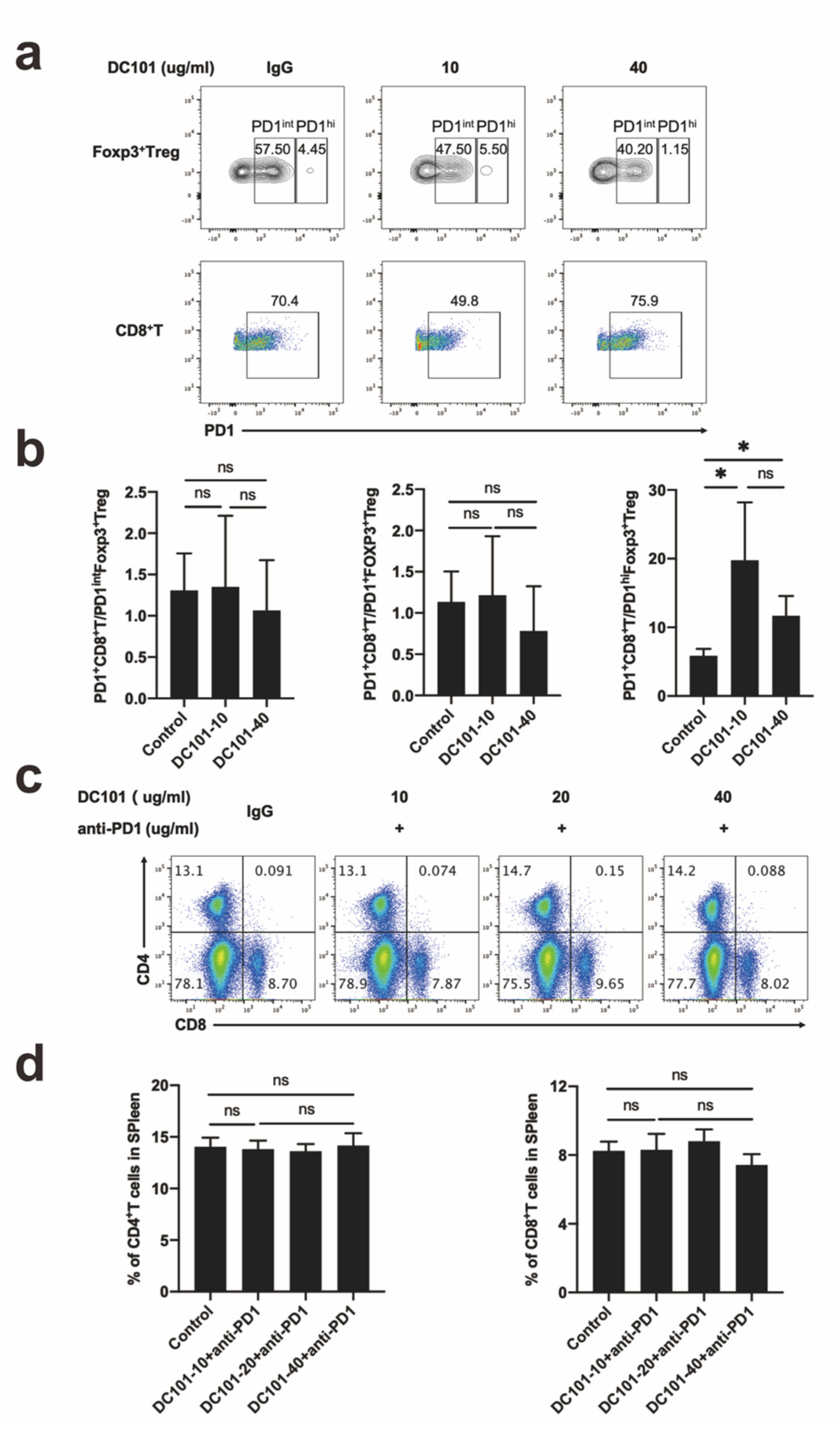
a
DC101 (ug/ml)
IgG
10
40
Foxp3+Treg
PD1int PD1hi
57.50
4.45
47.50
5.50
40.20
1.15
CD8+T
70.4
49.8
75.9
PD1
b
PD1+CD8+T/PD1intFoxp3+Treg
PD1+CD8+T/PD1+FOXP3+Treg
PD1+CD8+T/PD1hiFoxp3+Treg
ns
*
Control
DC101-10
DC101-40
c
DC101 (ug/ml)
anti-PD1 (ug/ml)
IgG
10
20
40
+
13.1
0.091
78.1
8.70
13.1
0.074
78.9
7.87
14.7
0.15
75.5
9.65
14.2
0.088
77.7
8.02
CD4
CD8
d
% of CD4+T cells in SPleen
% of CD8+T cells in SPleen
ns
Control
DC101-10+anti-PD1
DC101-20+anti-PD1
DC101-40+anti-PD1

Fig. S8 DC101 combined with anti-PD1 treated tumor-bearing mice, there was no statistical difference in the percentage of T cells in the spleen among the groups. (a, b) Mice were injected subcutaneously with LA795 cells to make a tumor-bearing mice model. They were given intraperitoneal injection of DC101 11 days after tumor cell inoculation. After all the administration of tumor-bearing mice was finished, analysis of tumor-infiltrating $PD1^{+}CD8^{+}T$ cells, expression of PD1 on tumor-infiltrating $Foxp3^{+}CD4^{+}T$ ($Foxp3^{+}Treg$) cells and the ratio of $PD1^{+}CD8^{+}T$ to $PD1^{int}Foxp3^{+}Treg$, the ratio of $PD1^{+}CD8^{+}T$ to $PD1^{+}Foxp3^{+}Treg$, the ratio of $PD1^{+}CD8^{+}T$ to $PD1^{hi}Foxp3^{+}Treg$ was performed by flow-cytometry. Representative histograms were present (n=3). (c, d). Mice were injected subcutaneously with LA795 cells to make a tumor-bearing mice model. They were given intraperitoneal injection of DC101 11 days after tumor cell inoculation and anti-PD1 antibody 12 days after tumor cell inoculation. After all the administration of tumor-bearing mice was finished, analysis of percentage of $CD8^{+}T$ and $CD4^{+}T$ cells in spleen was performed by flow cytometry. Representative histograms were present (n=5). One representative experiment out of three was shown.

Fig. S9

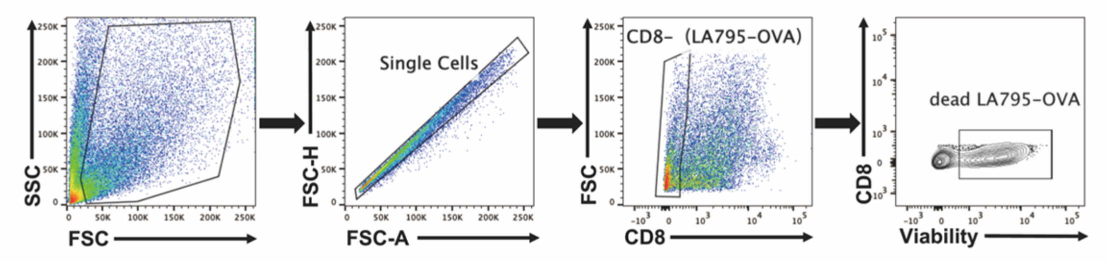

Fig. S9 Dead LA795-OVA cells Gating after being co-cultured with OTI $CD8^+$ T cells. After LA795-OVA cells were co-cultured with OTI $CD8^+$ T cells, both of them were harvested and mixed for analysis by flow cytometry. LA795 cells were gated on $CD8^-$ T cells, from which the dead LA795-OVA cells were gated.

Fig. S10

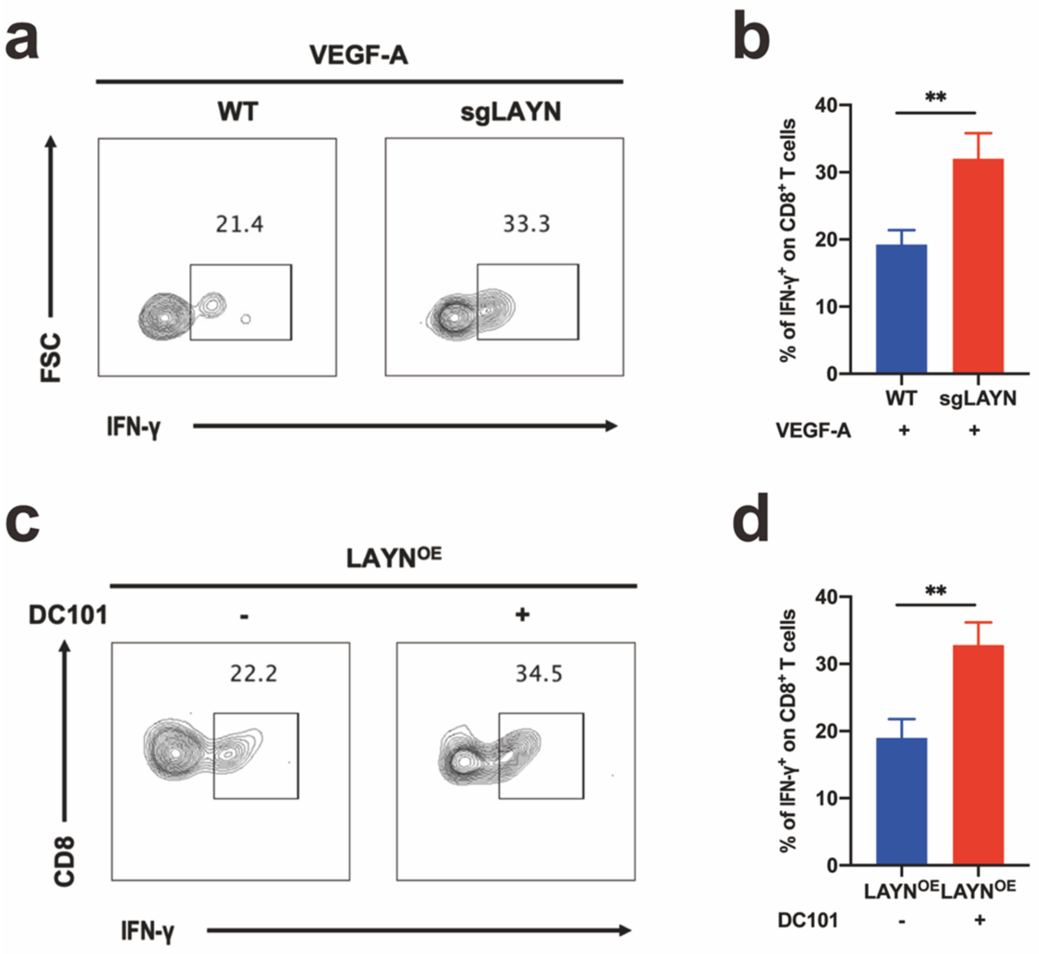

Fig. S10 VEGF-A affected CD8$^+$ T cell function through regulating LAYN expression. (a, b) WT and CD8$^+$ T cells knocked out LAYN were treated with VEGFA, and the function of CD8$^+$ T cells was analyzed by flow cytometry (n=4 per group). (c, d) CD8$^+$ T cells overexpressed LAYN were treated with DC101 (anti-VEGFR2) or not respectively, the function of CD8$^+$ T cells was analyzed by flow cytometry (n=4 per group). One representative experiment out of three was shown.

Fig. S11

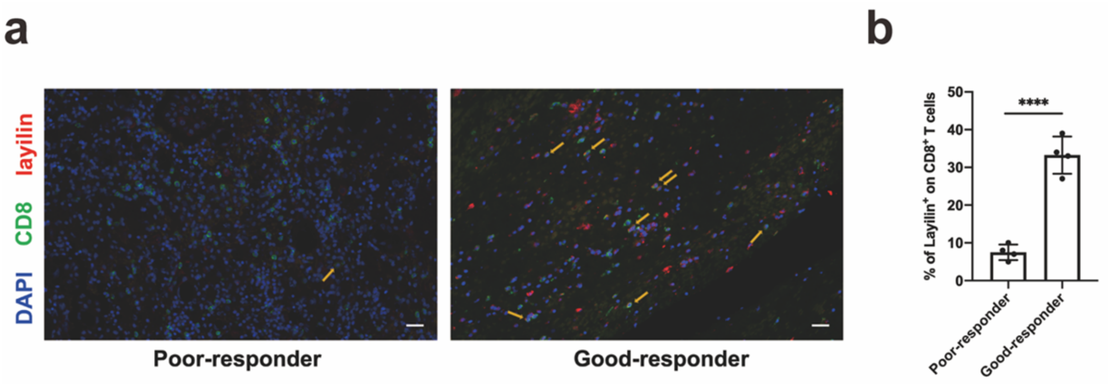

Fig. S11 Double positive fluorescence staining of CD8 and LAYN in the good-responder group was higher than that in the poor-responder group. (a, b) We performed double immunofluorescence staining of LAYN and CD8 on tumor tissue samples of the good-responder group and poor-responder group. (n=4 per group) (Scale bar, 25μm).
